# The protective role of nicardipine in dextran sulfate sodium-induced colitis in mice: Modulating inflammation and apoptosis

**DOI:** 10.1016/j.toxrep.2025.102123

**Published:** 2025-09-02

**Authors:** Ali M. Al-Joda, Munaf H. Zalzala

**Affiliations:** a Department of Pharmacology and Toxicology, College of Pharmacy, University of Baghdad, Baghdad 10047, Iraq; b Ministry of Health and Environment, Babil, Babylon 51001, Iraq

**Keywords:** Ulcerative colitis, Nicardipine, Cytokines, Inflammasomes, Apoptosis

## Abstract

Ulcerative colitis (UC) is a chronic inflammatory bowel disease associated with persistent inflammation, oxidative stress, and epithelial apoptosis. Nicardipine, a dihydropyridine calcium channel blocker, exhibits anti-inflammatory and anti-apoptotic properties, but its therapeutic potential in UC remains unclear. This study evaluated the effects of nicardipine on dextran sulfate sodium (DSS)-induced colitis in mice, focusing on inflammatory, oxidative, and apoptotic pathways. Fifty BALB/c mice were assigned to five groups (n = 10): control, DSS, nicardipine 12 mg/kg, nicardipine 24 mg/kg, and 5-aminosalicylate (ASA) 75 mg/kg. Treatments were administered for 3 days before and 10 days during DSS exposure. Disease severity was assessed by body weight, disease activity index (DAI), and colon length. Colonic mRNA levels of *Nlrp3*, *TNF-α*, *IL-17, and TNFSF10 were quantified by RT-PCR; protein expression of caspase-3, caspase-8, BAX, and BCL-2* was analyzed by Western blot. Serum malondialdehyde (MDA), myeloperoxidase (MPO), glutathione peroxidase-1 (GPX-1), occludin, and prostaglandin E₂ (PGE-2) were measured by ELISA. Histological scoring assessed epithelial integrity and inflammation. Nicardipine dose-dependently reduced DSS-induced weight loss, DAI, and colon shortening. Both doses significantly downregulated *Nlrp3*, *TNF-α*, *IL-17, and TNFSF10 (p < 0.05), decreased caspase-3 and BAX, and increased BCL-2*. Nicardipine restored GPX-1, lowered MDA and MPO, preserved occludin, and reduced PGE-2. Histology confirmed reduced mucosal injury and preserved epithelial architecture. Nicardipine attenuates DSS-induced colitis by suppressing pro-inflammatory cytokines, reducing oxidative stress, and inhibiting apoptosis, supporting its potential as a therapeutic candidate for UC. Further studies are warranted to clarify its molecular mechanisms and clinical relevance.

## Highlights

• Nicardipine dose-dependently reduces DSS-induced weight loss, DAI, colon shortening.
• Nicardipine suppresses colonic Nlrp3, TNF-α, IL-17 and TNFSF10 mRNA expression.
• Nicardipine reduces caspase-3 and Bax, elevates Bcl-2, inhibiting apoptosis.
• Nicardipine restores GPX-1, lowers MDA and MPO, and preserves occludin integrity.

## Graphical Abstract

**Figure:**
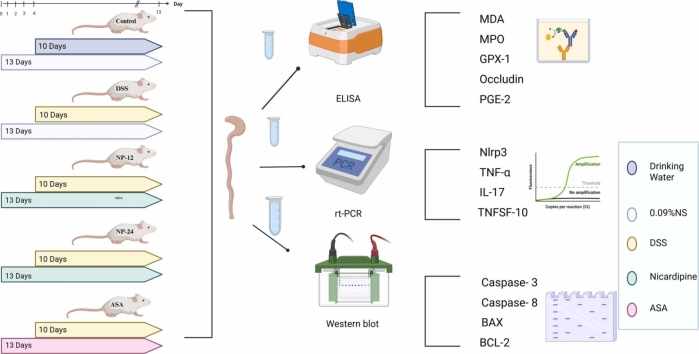

## 1 Introduction

Ulcerative colitis (UC) is a chronic inflammatory disease that affects the colon and rectum, characterized by mucosal inflammation that typically starts in the rectum and can potentially spread. Symptoms include hematochezia, abdominal pain, and urgent defecation. Its global prevalence is increasing [1]. The etiology of UC is multifactorial, involving a complex interplay of environmental, genetic, and lifestyle-related factors [2]. Among the significant risk factors associated with the onset and progression of UC are dietary patterns, tobacco use, hereditary susceptibility, and the influence of pharmacological interventions. These factors collectively contribute to the disease's pathogenesis and its associated clinical complications [3].

Apoptosis is a tightly regulated process critical for maintaining tissue equilibrium by eliminating dysfunctional or excess cells. It is characterized by distinctive structural and molecular properties that are crucial for both healthy functioning and disease state [4]. Apoptosis drives UC progression by disrupting intestinal epithelial cells (IECs), increasing permeability, and promoting inflammation. UC patients exhibit heightened IEC apoptosis, resulting in barrier dysfunction and chronic disease [5]. In animal models, oral DSS induces colitis resembling UC and may lead to colon cancer with prolonged exposure. However, its pathogenic mechanisms, especially regarding epithelial apoptosis and proliferation, remain unclear. DSS-induced acute colitis in mice is characterized by increased apoptosis and cell cycle arrest in colonic epithelial cells [6].

DSS-induced colitis is closely associated with ROS production, which worsens inflammation and colon damage. Research shows that ROS are generated intracellularly and extracellularly, highlighting oxidative stress as a key factor in disease progression [7]. ROS-induced lipid peroxidation drives apoptosis by damaging bio-membranes, triggering chain reactions that lead to cell death via apoptosis, autophagy, and ferroptosis. This mechanism is widely conserved across various cell types and conditions [8]. Mitochondria, as key producers of reactive oxygen species (ROS), initiate apoptosis through the release of cytochrome-C and caspase activation. Elevated ROS compromises mitochondrial membrane integrity, enhancing pro-apoptotic signaling (Bax, Bak) and suppressing Bcl-2. Cytochrome c, once in the cytosol, engages APAF-1, leading to the activation of caspases 9 and 3. ROS also activates the Nlrp3 inflammasome, linking oxidative stress to inflammatory cell death [9], [10].

The Nlrp3 inflammasome drives inflammation by activating IL-1β and IL-18, making it a potential therapeutic target in UC. Interleukin-1 beta (IL-1β) enhances tumor necrosis factor-alpha (TNF-α) signaling, inducing apoptosis via the TRAIL/TNFSF pathway and nuclear factor kappa B (NF-κB) activation. In DSS colitis, NLRP3-driven oxidative stress upregulates Bax and downregulates Bcl-2, thereby promoting apoptosis [11]. Interleukin-17 (IL-17) has been implicated in the activation of the Nlrp3 inflammasome across multiple cellular contexts and pathological conditions. Specifically, in retinal pigment epithelial (RPE) cells, IL-17A stimulates the release of IL-1β through a mechanism that engages Nlrp3 inflammasome assembly. This process is mediated by signaling cascades, including NF-κB and mitogen-activated protein kinases (MAPKs), and is further amplified by the production of reactive oxygen species (ROS). It is implicated in autoimmune diseases like rheumatoid arthritis, psoriasis, and inflammatory bowel disease. Given its role in immune dysregulation, IL-17 is a potential therapeutic target. In active UC, elevated IL-17 protein and mucosal mRNA levels suggest its critical involvement in disease pathogenesis [12], [13]. Nicardipine, a dihydropyridine calcium channel blocker (CCB), is used to treat hypertension and angina. It selectively inhibits L-type calcium channels, promoting arterial vasodilation and lowering blood pressure [14]. Studies demonstrate that L-type selective CCBs regulate physiological processes and exhibit anti-inflammatory effects by modulating inflammatory pathways and reducing cytokine production [15]. Multiple studies have demonstrated that NP exerts anti-inflammatory effects and mitigates oxidative stress, resulting in decreased inflammation and tissue damage by regulating inflammatory pathways and cytokine release [16].

This study is the first to comprehensively demonstrate that nicardipine, a dihydropyridine calcium channel blocker, exerts multi‑target protective effects in dextran sulfate sodium–induced ulcerative colitis by simultaneously modulating inflammatory cytokines (TNF‑α, IL‑17, TNFSF‑10), suppressing NLRP3 inflammasome activation, attenuating oxidative stress, and restoring epithelial barrier integrity. Unlike previous reports that focused on the general anti‑inflammatory or antioxidant properties of calcium channel blockers, our work uniquely elucidates nicardipine’s coordinated regulation of apoptosis (via caspase‑3, caspase‑8, Bax, and Bcl‑2) and tight junction preservation (occludin) in the context of colonic inflammation. This integrated mechanistic insight positions nicardipine as a novel therapeutic candidate for ulcerative colitis, bridging a critical knowledge gap between calcium channel modulation and intestinal immune‑epithelial homeostasis. This study investigates the therapeutic effects of nicardipine on DSS-induced colitis in mice, with a focus on inflammation, oxidative stress, apoptosis, and mechanisms that preserve the intestinal barrier.

## 2 Materials and methods

### 2.1 Chemicals and reagents

DSS (Dextran Sulfate Sodium) with a molecular weight of 40,000 and a purity exceeding 98 %, along with nicardipine hydrochloride and RNAlater, were obtained from Sigma Aldrich (USA). Polyclonal primary and secondary antibodies specific to target proteins, including BCL-2, Caspase-3, Caspase-8, BAX, and β-actin, as well as buffers and reagents such as the western blot detection kit and SDS-PAGE gel kit, were sourced from Elabscience Biotechnology Inc. (Wuhan, China). The RNA extraction kit and master mix were purchased from Promega Corporation (Madison, WI, USA). OneScript® Hot Reverse Transcriptase was acquired from Applied Biological Materials Inc. (ABM) (Canada). Primers for target genes (TNF-α, IL-17, Nlrp3, TNFSF, and IL-10) utilized for RT-PCR analysis were supplied by Macrogen, Inc. (South Korea). ElISA kits were sourced from Elabscience Biotechnology Inc. to evaluate MDA, MPO, occludin, GPX-1, and PGE-2 levels.

### 2.2 Animal housing, animal care, and ethical considerations

Fifty male healthy adult BALB/c albino mice aged 6 – 10 weeks, weighing 20 – 30 g, were obtained from an animal house in the College of Pharmacy, University of Baghdad. The experimental animals were housed in an environment with a regulated 12-hour light/dark cycle, a temperature of 22 ± 2 °C, and 45–50 % humidity. Mice had free access to standard rodent feed and tap water.

The study was approved by the Research Ethics Committee of the University of Baghdad, College of Pharmacy, with approval number “RECO2202512A” dated May 7, 2024. The study follows the framework of the Office International ‎Des ‎Épizooties’ principles on animal ethics guidelines.‎ The methods used on the animals fully comply with regional and international regulations governing the ethical treatment and use of laboratory animals. ‎The authors complied with the ARRIVE 2.0 guidelines [17].

Before starting the experiment, the animals were given a week to acclimate to the laboratory conditions. Throughout the experiment, mice were subjected to daily observations for signs of distress or illness, and their body weights were consistently recorded to monitor their overall health. For mice, body weight was monitored and documented daily during this period. Furthermore, stool consistency and rectal bleeding were assessed daily. The disease activity index (DAI), which incorporates changes in body weight, stool consistency, and rectal bleeding in mice, was calculated and recorded daily.

### 2.3 Experimental design

**Fig. 1:**
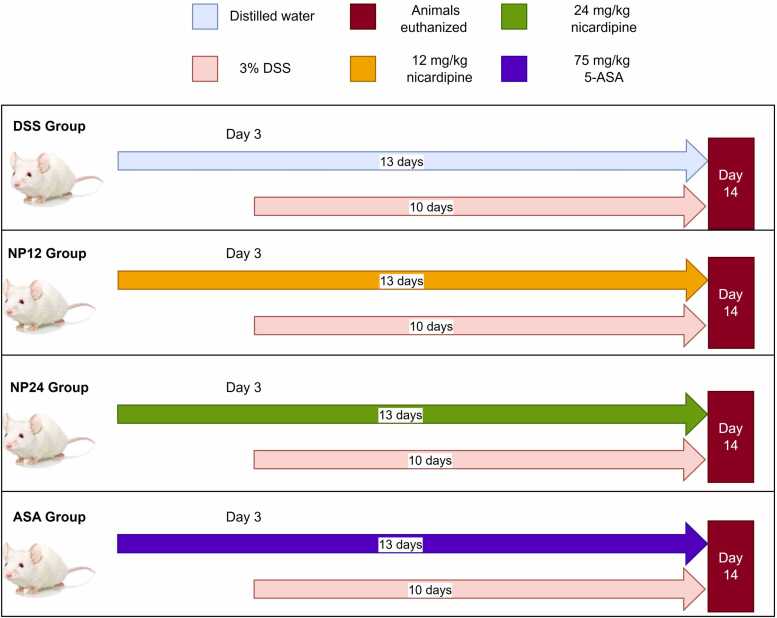
Flow chart of the study.

After the acclimatization period, the mice were randomly divided into five groups (n = 10), including the normal control group (Control group) and the DSS-induced UC model group (DSS group), which was administered orally via a cage feeding bottle at a concentration of 3 % for 10 days [18]. Two groups receiving nicardipine treatment were administered a single daily dose of 12 mg/kg orally via gastric gavage for three days before starting DSS feeding and then alongside DSS for ten days (NP12 group). The second group will receive a single daily dose of 24 mg/kg in the same manner (NP24 group) [19], [20]. The comparison group (ASA group) received 5-amino salicylic acid (ASA) in a dose of 75 mg/kg orally administered by gastric gavage 3 days before starting feeding DSS and then concomitant with DSS for 10 days [21], as seen in Fig. 1.

All mice were monitored daily for body weight and clinical observations. On the tenth day, Animals were anesthetized with isoflurane, following approved ethical protocols. Following euthanasia (the mice were anesthetized intraperitoneally with 80 mg/kg of ketamine and 10 mg/kg of xylazine; after complete anesthesia, the mice were euthanized by cervical dislocation) [22]. A portion of the colon was preserved in 4 % paraformaldehyde for histological analysis. In contrast, a portion of tissue was rapidly kept in RNAlater (Sigma-Aldrich, USA) for real-time PCR, and another portion was rapidly frozen at −80 °C for western blot studies [23].

### 2.4 Disease activity index (DAI)

The DAI serves as a crucial parameter in experimental models of UC, offering valuable insights into disease severity and therapeutic efficacy. This index incorporates critical indicators, including body weight reduction, stool consistency, and the presence of rectal bleeding, which are fundamental for assessing the progression and management of UC in murine models. The evaluation and scoring of colitis were conducted using DAI, based on parameters such as weight loss, diarrhea, and rectal bleeding [24].

### 2.5 Histopathological analysis

Colon tissues from mice were fixed in 10 % neutral buffered formalin, processed, and embedded in paraffin. Sections (4 μm) were stained with hematoxylin to highlight nuclei and eosin for cytoplasm visualization. The slides were dehydrated, cleared, and mounted with resin for histopathological analysis under a light microscope. An independent observer conducted a blinded histopathological assessment of colonic tissues. Scoring was performed based on a composite assessment of predefined criteria, including the severity of epithelial injury, crypt architecture disruption, immune cell infiltration, and the presence of submucosal edema [25].

### 2.6 Western blot analysis

Protein extraction and Western blotting were conducted using the Elabscience® kit protocol. Colonic tissues were homogenized in RIPA buffer containing protease and phosphatase inhibitors. Lysates were incubated on ice (30 min) and centrifuged (12,000 × g, 15 min, 4°C) to remove debris. Supernatants were collected, and protein concentrations were determined via BCA assay. SDS-PAGE separates 50 μg of protein, transfers it to PVDF membranes, and blocks with 5 % non-fat milk or BSA in TBST for 1 h at room temperature. Membranes were incubated overnight at 4°C with primary antibodies, followed by HRP-conjugated secondary antibodies (1 h, RT). Bands were detected using ECL and imaged with a Bio-Rad ChemDoc system. Protein expression was quantified using ImageJ, normalized to β-actin or GAPDH [26].

### 2.7 Enzyme-linked immunosorbent assay (ELISA)

Serum levels of malondialdehyde (MDA; Cat# E-El-0060), myeloperoxidase (MPO; Cat# EM0010), occludin (cat# E-EL-H1073, glutathione peroxidase-1 (GSH1; Cat# E-EL-M0950), and prostaglandin E2 (PGE-2; E-EL-0034) were quantitatively determined using specific enzyme-linked immunosorbent assay (ELISA) kits, following the standardized protocols provided by Elabscience (Wuhan, China).

### 2.8 Reverse transcription polymerase chain reaction (RT-PCR)

Total RNA was isolated from colon tissue samples utilizing the SV Total RNA Isolation System (Promega, USA), strictly adhering to the manufacturer’s standardized protocol to ensure high-quality RNA extraction. The concentration and purity of the extracted RNA were quantitatively and qualitatively assessed using a Nanodrop spectrophotometer (Microdigital, South Korea), ensuring an optimal A260/A280 ratio for downstream applications. Subsequently, complementary DNA (cDNA) synthesis was performed using 1 µg of total RNA as a template, employing the OneScript Hot Reverse Transcriptase enzyme according to the provided procedural guidelines. This step was carried out under precise thermal cycling conditions to maximize cDNA yield and integrity. For the quantification of gene expression, real-time quantitative polymerase chain reaction (RT-qPCR) was conducted using the CFX96 real-time PCR system (Bio-Rad, USA). Reactions were performed in a total volume of 20 µL, comprising the synthesized cDNA, gene-specific primers, and SYBR Green Master Mix as the fluorescent detection reagent. The housekeeping gene GAPDH was employed as an internal reference to normalize expression levels and account for potential variations in RNA input or reverse transcription efficiency. Relative quantification of gene expression was determined using the 2^-ΔΔCt^ method. The primers utilized in this study were meticulously designed using Primer Design Software. The specific sequences of the primers used in this study are detailed in Table 1.

**Table ‎1:** Primer sequences for RT-PCR.

**Gene**	**Forward primer (5′–3′)**	**Reverse primer (5′–3′)**	**Accission No.**
Nlrp3	TACGGCCGTCTACGTCTTCT	CGCAGATCACACTCCTCAAA	XM_036156548.1
TNF-α	GCCTCTTCTCATTCCTGCTTG	CTGATGAGAGGGAGGCCATT	NM_001278601.1
TNFSF10	CTGTGTCTGTGGCTGTGACT	GTCTTCCACCTCTGGGCAAG	NM_009425.2
IL−17	TCCAGAATGTGAAGGTCAACC	TATCAGGGTCTTCATTGCGG	NM_010552.3
IL−10	ACTGCTATGCTGCCTGC	CACCTTGGTCTTGGAGC	NM_010548.2
GAPDH	CTTTGTCAAGCTCATTTCCTGG	TCTTGCTCAGTGTCCTTGC	NM_001411842.1

### 2.9 Statistical analysis and sample size calculation

The animal sample size was determined using the G.Power 3.1 software (*post hoc* sample size was done with an effect size of 0.53 and an alpha level of 0.05, F-family tests with a total sample size of 50, with 10 animals per group) [27].

Tukey’s Honestly Significant Difference test was used for controlling multiple comparisons (because it provides a controlled and systematic way to compare all possible pairs of group means while protecting against the increased risk of type I errors that arise with multiple comparisons). Alternatively, if the variables did not follow a normal distribution, we applied the Dunn test to control for multiple comparisons. This rank-based approach makes it more appropriate when dealing with skewed distributions or when outliers might distort the results of parametric tests. Additionally, like other post hoc methods, Dunn’s test incorporates adjustments—often via methods like the Bonferroni or Sidak corrections—to control the familywise error rate, ensuring that the likelihood of making one or more type I errors across all tests stays within the targeted significance. GraphPad Prism version 10.4.1 for Windows, San Diego, California, USA, was used to perform the statistical analysis. The p-value was considered significant if less than 0.05.

## 3 Results

### 3.1 Nicardipine mitigated the effects of DSS-induced experimental colitis

**Fig. 2:**
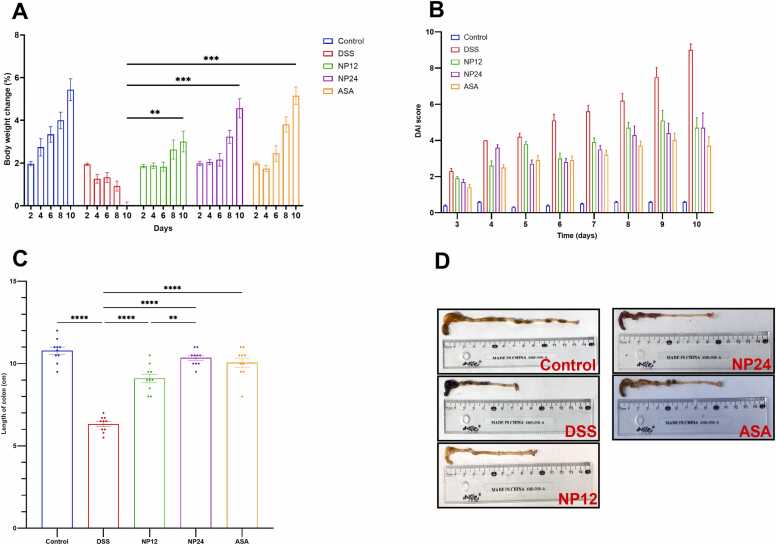
A) illustrates the percentage change in body weight, with mice being monitored and their body weight being recorded every two days following DSS administration. (B) The Disease Activity Index (DAI) was assessed based on body weight fluctuations, stool consistency, and rectal bleeding. (C) Colon length measurements across experimental groups. (C) Colon images of different groups. ** indicates p < 0.01, **** indicates p < 0.0001. Data are expressed as mean ± SEM.

A 10-day DSS administration caused acute colitis, evidenced by a reduction in body weight beginning on day 4 compared to the control group (Fig. 2A). Both doses of NP, 12 mg/kg (NP-12) and 24 mg/kg (NP-24), suppressed DSS-induced body weight loss starting on day 4 and lasting up to day 10. NP-24 is more effective than NP-12 in mitigating the effects of DSS-induced experimental ulcerative colitis on body weight, as seen in Fig. 2A

Mice with colitis also exhibited symptoms such as loose stools and hematochezia, prompting the evaluation of the DAI. Treatment with NP-24 notably reduced this high score more than NP-12 (Fig. 2B). NP-24 demonstrated greater efficacy than NP-12 in alleviating DSS-induced ulcerative colitis.

Furthermore, the DSS-UC mice model is typically characterized by a substantial reduction in colon length, a well-established marker of colonic inflammation and disease severity (Figs. 2C and 2D). Notably, pre-treatment with NP at doses of 12 mg/kg and 24 mg/kg, as well as ASA at 75 mg/kg, significantly mitigated these pathological manifestations. Mice receiving these interventions exhibited a notable preservation of body weight and a significant restoration of colon length when compared to the DSS-induced colitis group, highlighting the therapeutic potential of these compounds in attenuating colonic inflammation and structural deterioration.

### 3.2 NP Mitigates DSS-induced histopathological alterations and colonic infiltration

As illustrated in Fig. 2A, the colonic tissues of the control group exhibited an intact and well-organized histological architecture characterized by well-preserved crypt structures and a continuous epithelial lining. In contrast, the DSS-induced colitis group demonstrated pronounced histopathological damage, including extensive goblet cell depletion, mucosal necrosis, and severe architectural distortion or complete loss of crypt epithelium, accompanied by substantial infiltration of inflammatory cells (Fig. 2B). However, pre-administration of NP at doses of 12 mg/kg and 24 mg/kg, as well as ASA at 75 mg/kg, markedly attenuated these pathological alterations, as shown in Fig. 2C, D, and E, respectively. Histological assessment revealed that NP and ASA treatment contributed to the preservation of crypt morphology and maintenance of colonic mucosal integrity, thereby mitigating the severity of DSS-induced colonic damage. These observations were supported by histological scoring (2 F), which revealed a significant elevation in damage scores in the DSS group compared to the control (p < 0.001). In contrast, all treatment groups showed statistically significant reductions in histological scores relative to DSS (p < 0.001), confirming the protective efficacy of NP and ASA against DSS-induced colonic injury.

### 3.3 NP suppressed apoptosis in the mice with DSS-induced colitis

**Fig. 3:**
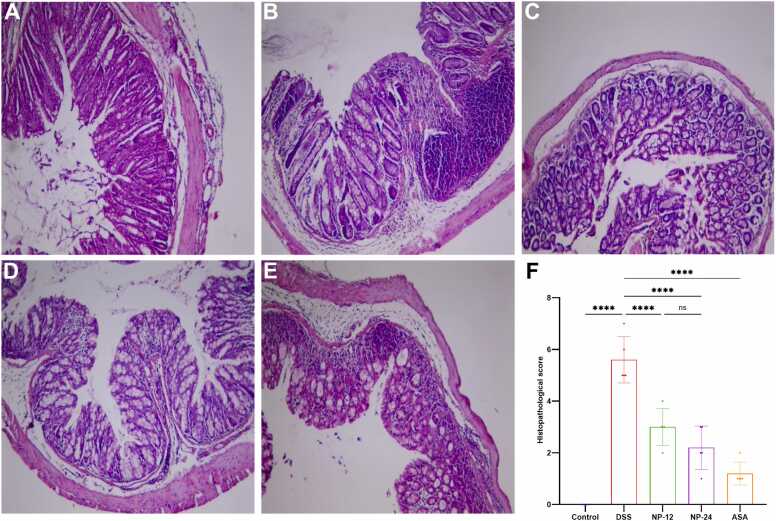
Histological Analysis of Colonic Tissue: Representative histological images illustrate the structural characteristics of colonic tissue across different experimental groups. Panel (A) shows normal colonic architecture in the control group, whereas panel (B) shows the DSS-colitis model. Panels (C) colonic architecture of Np administration at a dose of 12 mg/kg and (D) NP at a dose of 24 mg/kg. Panel (E) represents the colonic tissue from the ASA treatment group. All images were captured at an original magnification of 40X. Panel (E) represents the histological score (data presented as mean ± SEM), **** indicates p < 0.0001.

The levels of key apoptosis-related proteins were evaluated using Western blot analysis (Fig. 3A), showing significant changes in the DSS-induced colitis model. DSS administration markedly increased the expression of pro-apoptotic proteins, including Caspase 3, Caspase 8, and Bax, while concurrently reducing the level of the anti-apoptotic protein Bcl-2, compared to the control. Quantitative analysis (Fig. 3B–E) revealed significant upregulation of Caspase 3 and Caspase 8 in the DSS group, indicating activation of both the intrinsic and extrinsic apoptotic pathways. Bax expression was also significantly elevated, further supporting enhanced pro-apoptotic signaling. Conversely, Bcl-2 was significantly downregulated in the DSS group, suggesting a compromised anti-apoptotic defense. Treatment with NP at both treated doses attenuated the expression of pro-apoptotic markers and restored Bcl-2 levels in a dose-dependent fashion, with NP-24 showing more pronounced effects. The ASA-treated group exhibited a comparable regulatory pattern.

### 3.4 Oxidative stress biomarkers are attenuated by NP treatment in DSS-induced colitis

**Fig. 4:**
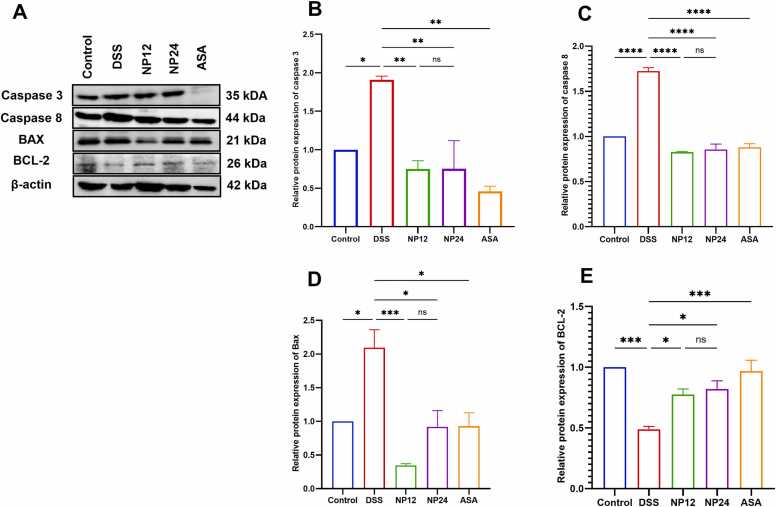
Effects of treatments on apoptotic markers expression in DSS-induced colitis model. (A) Western blot images. (B) Relative protein expression of caspase 3. (C) Relative protein expression of Caspase 8. (D) Relative protein expression of Bax. (E) Relative protein expression of BCL-2. The expression levels of these genes were quantified using Western blot, expressed as a percentage relative to Beta-actin mRNA levels. Statistical significance is denoted as follows: * p < 0.05, ** p < 0.01, *** p < 0.001; **** P < 0.0001. Data are presented as mean ± SEM.

DSS exposure significantly changed serum oxidative stress markers, increasing malondialdehyde (MDA) and myeloperoxidase 9MPO), while decreasing glutathione peroxidase-1 (GPX-1) activity (Figs. 4A to 4C). Specifically, DSS treatment induced a sharp rise in MDA, indicative of extensive lipid peroxidation, and a parallel increase in MPO levels, reflecting neutrophil activation and oxidative burden. Conversely, GPX-1 levels were substantially suppressed in the DSS group relative to the control, suggesting a compromised endogenous antioxidant defense system. Administration of NP at 12 and 24 mg/kg significantly reversed these changes, with both doses restoring GPX-1 levels and reducing MDA and MPO concentrations in a dose-responsive manner. Notably, NP-24 demonstrated superior efficacy, closely approximating the antioxidant profile observed in the ASA-treated group. These findings underscore the capacity of NP to counteract oxidative tissue damage by modulating key redox-sensitive enzymes and limiting ROS-mediated injury in colitic mice.

### 3.5 NP restores epithelial barrier integrity via modulation of occludin and PGE-2 levels

To evaluate epithelial barrier disruption during colitis and its restoration by treatment, serum levels of occludin and prostaglandin E2 (PGE-2) were measured in Figs. 4D and 4E, respectively. DSS exposure resulted in a notable decrease in occludin concentration, indicating the disassembly of tight junctions and a compromise of the mucosal barrier. Concurrently, there was a substantial increase in serum PGE-2 levels, demonstrating elevated inflammatory signaling and epithelial stress. NP administration significantly mitigated these alterations, with both doses preserving occludin expression and reducing PGE-2 concentrations compared to the DSS group. The higher dose NP-24 showed more pronounced restoration of epithelial integrity, comparable to the effect of ASA. These data suggest that NP exerts a protective effect on the intestinal barrier by preserving tight junction proteins and suppressing pro-inflammatory lipid mediators, thereby maintaining mucosal homeostasis under inflammatory conditions.

### 3.6 Nicardipine effect on the expression of pro-inflammatory cytokine levels and TNFSF-10 in the DSS-UC model

**Fig. 5:**
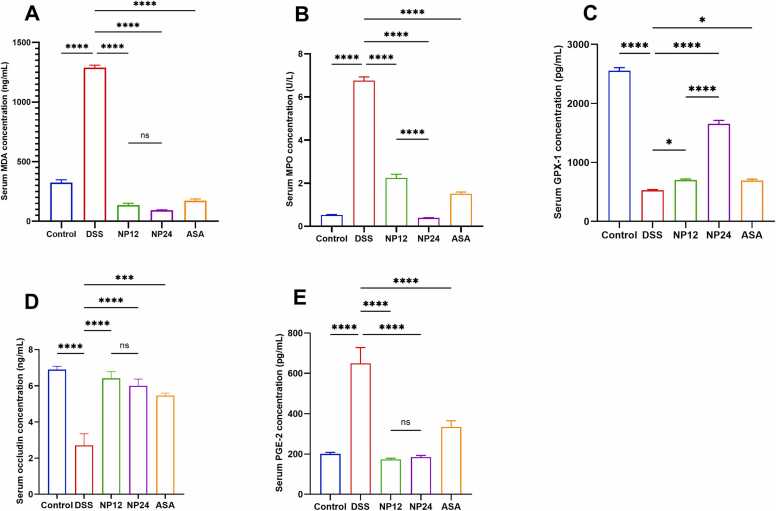
ELISA Analysis of Oxidative Stress, Inflammatory, and Barrier-Related Markers in Serum. (A) Represent serum MDA concentration (ng/mL) across experimental groups. (B) Represent serum MPO concentration (U/L) across experimental groups. (C) Represent Serum GPX-1 concentration (pg/mL) across experimental groups. (D) Represent Serum Occludin concentration (ng/mL) across experimental groups. (E) Represent Serum PGE-2 concentration (pg/mL) across experimental groups. Statistical significance is denoted as follows: * p < 0.05; **** P < 0.0001. Data are presented as mean ± SEM.

The expression levels of TNF-α, IL-17, and TNFSF-10 mRNA were analyzed across experimental groups using GAPDH as a reference gene for normalization. As illustrated in Fig. 5A, TNF-α expression was markedly elevated in the DSS-induced colitis group relative to the untreated control group, indicating a pronounced inflammatory response. Administration of NP at 12 mg/kg and 24 mg/kg, as well as ASA, significantly attenuated TNF-α expression compared to the DSS group. A similar pattern was observed for IL-17 and TNFSF-10, as shown in Figs. 5B and 5C, respectively. DSS treatment resulted in a substantial upregulation of both cytokines relative to controls, further confirming the presence of inflammation. Notably, treatment with NP at both dose levels and ASA significantly downregulated the expression of IL-17 and TNFSF-10, with the higher NP dose (24 mg/kg) exerting a more pronounced suppressive effect.

### 3.7 Effect of nicardipine on Nlrp3 inflammasome activation in DSS-induced colitis

**Fig. 6:**
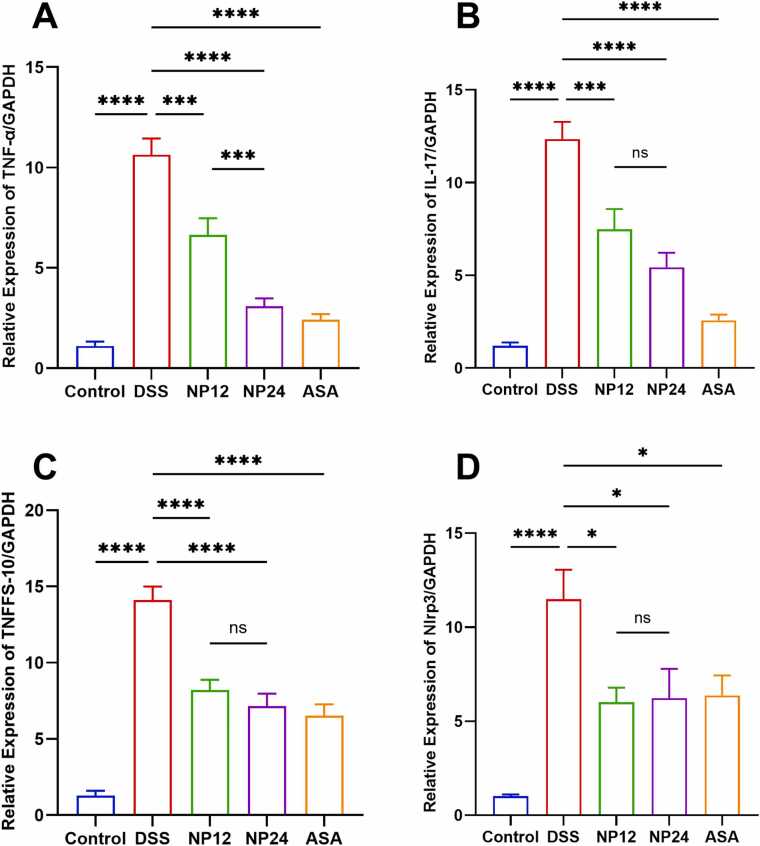
Effects of treatments on pro-inflammatory and apoptotic markers in the DSS-induced colitis model. (A) Relative expression of TNF-α across different experimental groups. (B) Relative expression of IL-17. (C) Relative expression of TNFSF10 (TRAIL). (D) Relative expression of Nlrp3. Statistical significance is denoted as follows: * p < 0.05, *** p < 0.001; **** P < 0.0001. Data are presented as mean ± SEM.

The Nlrp3 inflammasome, a key component of the innate immune system, plays a crucial role in the pathogenesis of UC. Upon activation by pathogen-associated molecular patterns and damage-associated molecular patterns, Nlrp3 assembles a multiprotein complex that activates Caspase-1, subsequently facilitating the maturation and secretion of the pro-inflammatory cytokines IL-1β and IL-18. This process amplifies inflammation and contributes to mucosal damage in UC [28]. As depicted in Fig. 5D, a significant upregulation of Nlrp3 mRNA expression was observed in the DSS-induced colitis model compared to the control group, further supporting its role in colonic inflammation. Notably, pre-treatment with NP at doses of 12 mg/kg and 24 mg/kg, along with ASA (75 mg/kg), effectively attenuated the inflammatory response by significantly downregulating Nlrp3 mRNA expression compared to the DSS-UC model. These findings strongly suggest that NP confers a protective effect in DSS-induced colitis by modulating Nlrp3 inflammasome activation, thereby reducing the production of pro-inflammatory cytokines and mitigating intestinal inflammation. This highlights the potential therapeutic implications of NP in targeting Nlrp3-mediated inflammatory pathways, which could contribute to improved treatment strategies for UC. (Fig. 6)

## 4 Discussion

Ulcerative colitis is a chronic IBD characterized by mucosal inflammation, epithelial cell apoptosis, and barrier dysfunction, primarily affecting the colon and rectum, leading to disease progression [29]. This study revealed a significant reduction in animal body weight and pathological changes in the colon. A shortened colon length, indicative of tissue edema and inflammatory infiltration, characterized these pathological changes. Additionally, the colon exhibited disruption of intestinal gland architecture, widespread infiltration of inflammatory cells, and hemorrhagic lesions, suggesting severe mucosal damage [30]. Administration of NP at dosages of 12 mg/kg and 24 mg/kg significantly ameliorated the observed pathological alterations, as indicated by the recovery of body weight and colon length in the animals. This suggests a potential protective role for NP in maintaining colonic integrity.

Apoptosis is a critical contributor to the pathogenesis of UC, a chronic inflammatory bowel disease characterized by intestinal epithelial damage and persistent mucosal inflammation [31]. Apoptotic dysregulation in UC involves caspase 3, caspase 8, Bax, BCL-2, and TNFSF-10, disrupting epithelial integrity and immunity, highlighting therapeutic targets to restore homeostasis and reduce severity [32]. DSS exposure markedly upregulated pro-apoptotic markers, including cleaved caspase 3, caspase 8, and Bax, as observed in the Western blot analysis, alongside a concomitant high expression of TNFSF-10 and suppression of the anti-apoptotic protein BCL-2. These alterations are indicative of enhanced apoptotic activity within the colonic mucosa, contributing to epithelial barrier disruption. Notably, treatment with NP significantly reversed these apoptotic signatures, restoring BCL-2 expression and reducing caspase 3 and caspase 8 activation, thereby suggesting a cytoprotective role via the inhibition of both intrinsic and extrinsic apoptotic pathways. NP exerts protective effects in colitis by restoring the apoptotic balance, thereby preserving epithelial homeostasis and barrier integrity [33], [34]. In addition to its anti-apoptotic properties, NP also demonstrated significant anti-inflammatory effects [35].

Disruption of the intestinal epithelial barrier is a hallmark of colitis pathogenesis, often marked by diminished expression of tight junction constituents and elevated inflammatory mediators. Previous studies have demonstrated that DSS exposure leads to decreased levels of key tight junction proteins, such as occludin [36]. In the present study, serum analyses revealed a significant reduction in occludin levels following DSS administration, reflecting compromised epithelial cohesion and impaired barrier function. Simultaneously, the observed rise in PGE-2, a key eicosanoid involved in inflammatory amplification and epithelial stress, further confirmed the ongoing mucosal injury [37]. PGE-2 is markedly upregulated in DSS-induced colitis, serving as both a pro-inflammatory mediator and a mucosal repair factor. Its expression reflects the intensity of intestinal inflammation and epithelial stress [37]. Notably, treatment with NP conferred marked protection against these alterations. Restoration of occludin concentrations, particularly evident at the higher NP-24 dose, underscores the compound’s potential in preserving tight junction architecture. Additionally, the significant attenuation of PGE-2 levels suggests that NP may limit the synthesis of inflammatory lipid mediators, contributing to its mucosal protective profile. These findings collectively indicate that NP fortifies epithelial barrier integrity by sustaining junctional protein expression and dampening PGE-2-driven inflammatory cascades, thereby countering epithelial destabilization under colitic conditions.

The Nlrp3 inflammasome mediates intestinal inflammation in UC by sensing stress, promoting the secretion of IL-1β and IL-18, epithelial damage, and immune activation, and is upregulated in DSS-induced colitis [38]. The study demonstrated that NP12 and NP24 groups have anti-inflammatory properties in UC by inhibiting Nlrp3 inflammasome activation, a key driver of UC pathogenesis. This suppression reduces mucosal damage, immune infiltration, and cytokine release, suggesting that nicardipine has potential as a novel therapeutic strategy for controlling intestinal inflammation in UC [16].

**Fig. 7:**
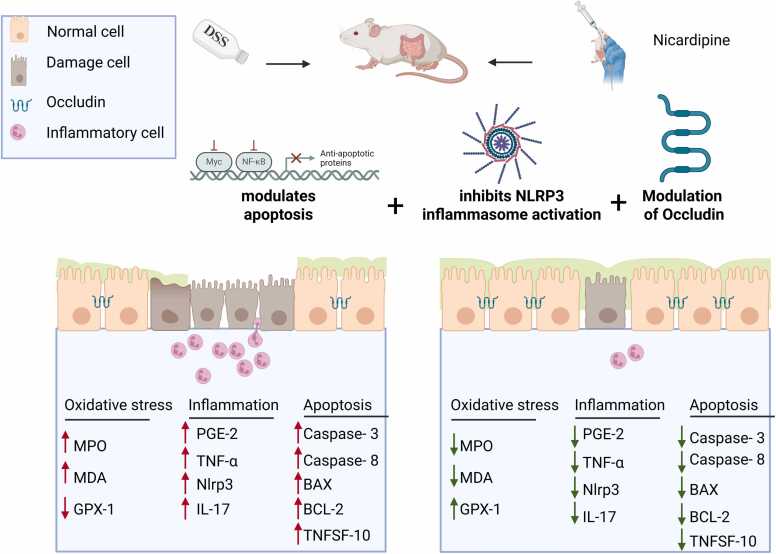
The Mechanistic scheme shows the effect of NP on DSS-induced ulcerative colitis: Nicardipine attenuates DSS-driven inflammatory and cell-death pathways: reduces TNF-α, IL-17, and TNFSF10 expression; limits NLRP3 priming/activation; decreases oxidative stress (lower MDA, MPO; higher GPX-1 vs DSS); downregulates pro-apoptotic markers (caspase-3, caspase-8, Bax) while upregulating BCL-2; and mitigates epithelial barrier disruption by attenuating occludin loss and reducing PGE-2. These changes are associated with partial prevention of weight loss, lower DAI, and reduced colon shortening compared with DSS.

Pro-inflammatory cytokines are key drivers in the development of UC, promoting persistent inflammation and leading to tissue damage within the intestinal mucosa [1]. TNF-alpha plays a crucial role in the inflammatory process of UC by driving mucosal inflammation and compromising the epithelial barrier. This disruption facilitates greater infiltration of immune cells, further intensifying inflammation. Additionally, TNF-alpha contributes to the apoptosis of epithelial cells, thereby weakening the intestinal lining and exacerbating disease progression. Its expression in colonic tissue was upregulated in the DSS-UC model [39], [40]. On the other hand, IL-17 is a key driver of DSS-induced colitis, promoting inflammation, neutrophil recruitment, and epithelial damage. IL-17A-deficient mice exhibit reduced weight loss, colonic injury, and mortality, highlighting its role in immune dysregulation. Targeting IL-17A may offer a therapeutic strategy for mitigating colitis-associated inflammation in IBD [41]. Oxidative stress is a hallmark of UC pathogenesis, characterized by elevated ROS and compromised antioxidant defense. The study revealed that DSS exposure significantly increased serum MDA and MPO levels—markers indicative of lipid peroxidation and neutrophil activation, respectively. Concurrently, GPX-1 activity was markedly suppressed, showcasing a disrupted endogenous antioxidant system. Fig. 7 summarizes the molecular mechanism of the nicradipine.

## 5 Conclusion

Our study shows that NP treatment reduces TNF-α and IL-17 levels in UC mice, indicating decreased inflammation. Nicardipine also modulates apoptosis and inflammasome pathways by increasing BCL-2 and decreasing caspase-3, which helps prevent epithelial damage. Additionally, it inhibits NLRP3 inflammasome activation, decreasing immune cell infiltration. These effects highlight NP's potential as a therapeutic agent to restore intestinal balance and reduce colonic injury in UC.

## Statements and declarations

None.

## CRediT authorship contribution statement

**Ali M. Al-Joda:** Writing – review & editing, Writing – original draft, Software, Resources, Methodology, Investigation, Formal analysis, Data curation, Conceptualization. **Munaf H. Zalzala:** Writing – review & editing, Writing – original draft, Visualization, Validation, Supervision, Methodology, Conceptualization.

## Consent to participate

Not applicable.

## Consent to publish

Not applicable.

## Ethics approval

“The study was approved by the Research Ethics Committee of the University of Baghdad, College of Pharmacy; all tests were conducted with approval number “RECO2202512A” dated May 7, 2024.” The methods used on the animals fully comply with regional and international regulations governing the ethical treatment and use of laboratory animals. ‎The authors complied with the ARRIVE 2.0 guidelines [17].

## Funding

The authors declare that no funds, grants, or other support were received during the preparation of this manuscript.

## Declaration of Competing Interest

The authors declare that they have no known competing financial interests or personal relationships that could have appeared to influence the work reported in this paper.

## Data Availability

Data will be made available on request.
